# Oophorectomy followed by postoperative chemotherapy for ovarian metastasis of colorectal cancer: a retrospective analysis

**DOI:** 10.3389/fonc.2025.1708323

**Published:** 2026-01-07

**Authors:** Guangyue Zhao, Yun Qiao, Yongchao Ba, Xing Song, Ye Tian, Yan Zhang, Ning Guan, Xiaoqian Hou, Yuhua Zhang

**Affiliations:** 1 Department of Colorectal Surgery, Cancer Hospital of China Medical University, Liaoning Cancer Hospital & Institute, Shenyang, Liaoning, China; 2 Department of Gastrointestinal Surgery, Changzhi People’s Hospital, Changzhi, Shanxi, China; 3 Breast Surgery, Cancer Hospital Affiliated to Harbin Medical University, Harbin, Heilongjiang, China; 4 Organ Transplantation Center, General Hospital of Northern Theater Command, Shenyang, Liaoning, China; 5 Second Department of Abdominal Oncology, Jilin Province Tumor Hospital, Changchun, Jilin, China; 6 Medical Examination Center, Cancer Hospital of China Medical University, Liaoning Cancer Hospital & Institute, Shenyang, Liaoning, China; 7 The Department of Breast Surgery, Liaoning Provincial Academy of Traditional Chinese Medicine (The Second Hospital Affiliated to Liaoning University of Traditional Chinese Medicine), Shenyang, Liaoning, China; 8 The Department of Gynecology, Cancer Hospital of China Medical University, Liaoning Cancer Hospital & Institute, Shenyang, Liaoning, China

**Keywords:** colorectal cancer, oophorectomy, ovarian metastasis, postoperative chemotherapy, prognostic factor

## Abstract

**Background:**

The incidence of ovarian metastasis from colorectal cancer is low but the prognosis is extremely poor; however, the prognostic factors remain unclear and there are currently no guidelines for the treatment of ovarian metastasis from colorectal cancer. This study evaluated the impact of resection of ovarian metastasis from colorectal cancer and postoperative chemotherapy on patient prognosis.

**Methods:**

This retrospective study analyzed patients with ovarian metastasis from colorectal cancer between January 2009 and December 2019. Factors with P < 0.1 in univariable analysis were considered as potentially prognostic factors and were incorporated into multivariable analysis with Cox proportional hazards regression models. Hazard ratios (HRs) with 95% confidence intervals (CIs) were analyzed. A two-sided P value < 0.05 was considered significant.

**Result:**

A total of 213 patients with ovarian metastasis from colorectal cancer were included in the study. Univariable analysis identified that the resection of primary tumor, extraovarian metastasis, oophorectomy, postoperative chemotherapy and mismatch repair (MMR) status as potentially prognostic factors. The median survival times in patients with and without oophorectomy were 25 and 11 months, respectively. Chemotherapy after surgery was associated with a longer median overall survival compared with patients without chemotherapy (25 versus 20 months). The median survival time for patients with dMMR status was 36 months, compared to 25 months for those with pMMR status. Multivariable analysis confirmed that oophorectomy (HR = 10.476, 95% CI, 5.536–19.825; P < 0.001), postoperative chemotherapy (HR = 2.232, 95% CI, 1.538–3.238; P < 0.001) and MMR status (HR = 1.967, 95% CI, 1.026-3.772; P = 0.042) were independent prognostic factors for overall survival in patients with ovarian metastasis from colorectal cancer.

**Conclusion:**

Oophorectomy, postoperative chemotherapy, and dMMR status may offer survival benefits for colorectal cancer patients with ovarian metastasis; however, the main findings from current study warrant further validations.

## 1 Introduction

Colorectal cancer (CRC) is one of the most common cancers worldwide, and both its morbidity and mortality have been increasing annually (1, 2). Advances in the comprehensive treatment of colorectal cancer have improved the cure rate and 5-year survival rate of patients with advanced colorectal cancer, but the prognosis remains poor (3–6).

CRC frequently metastasizes to distant organs such as the liver and lung, but recent advances in molecular-targeted agents and chemotherapy regimens have significantly improved the response rate and long-term survival (1–7). In contrast, although the incidence of ovarian metastasis from colorectal cancer (CROM) is low, at about 1.6%–10% (8–13), ovarian metastasis (OM) is insensitive to radiotherapy and chemotherapy, and the patient prognosis is thus very poor, with a median survival of 10.0–35.0 months and a 5-year survival rate of approximately 0%–26.6% (14–16).

In this study, we aimed to analyze the clinicopathological characteristics of CROM and to evaluate the prognostic significance of menopausal status, timing of OM, the status of extraovarian metastasis, resection of extraovarian metastasis, oophorectomy, and postoperative chemotherapy. We also aimed to identify independent prognostic factors for overall survival (OS) among patients with CROM.

## 2 Materials and methods

### 2.1 Patients

The present study evaluated patients with CROM treated at three hospitals between January 2010 and December 2019. All patients included in the study were completely anonymized. The study was conducted in accordance with the Declaration of Helsinki and approved by the Institutional Review Boards. The study results were reported according to the STROCSS guidelines (17). The inclusion criteria were as follows: (1) pathologically confirmed stage IV colorectal cancer; (2) histologically confirmed OM between January 2001 and December 2019; (3) informative pathology data (including pathological T and N stage, histology types, differentiation and immunohistochemistry data); (4) postoperative survival > 1 month; and (5) good performance status (Eastern Cooperative Oncology Group score of 0,1 or 2). The exclusion criteria were: (1) additional malignant neoplasms; and (2) postoperative survival ≤ 1 month; (3) incomplete data or loss to follow-up. The following baseline characteristics of the primary tumors and OM were collected: age, menopausal status, carcinogenicity antigen (CEA), CA199, tumor location, T and N stage according to the 8th edition of the American Joint Committee on Cancer Staging Manual, histology types, differentiation, timing of OM, side of metastasis, clinical symptoms, vascular endothelial growth factor (VEGF) status, epidermal growth factor receptor (EGFR) status, P53 status and mismatch repair (MMR) status. Patients with OM discovered 3 months after the first treatment were diagnosed with metachronous OM.

### 2.2 Outcome indicators

OS was identified as the primary outcome indicator, which was defined as the time from pathological diagnosis of colorectal cancer to death from any cause or last follow-up (18).

### 2.3 Statistical analysis

Univariable and multivariable Cox proportional hazard regression were conducted to identify independent prognostic factors for OS. Factors with *P* < 0.1 in univariable analysis were incorporated into multivariable analysis. Hazard ratios (HRs) with 95% confidence intervals (CIs) were analyzed. To further validate the robustness of our findings, we conducted stratified multivariable regression analysis based on primary tumor location (rectum or colon), pathological T stage (stage 1–3 or stage 4), pathological N stage (stage 0 or stage 1-2), and histology types (adenocarcinoma, mucinous adenocarcinoma, or signet-ring cell carcinoma). All analyses were performed using SPSS version 22.0 (SPSS Inc., Chicago, IL, USA) and R version 4.2.2 (R Foundation for Statistical Computing, Vienna, Austria) software. A two-sided P value < 0.05 was considered statistically significant.

## 3 Results

### 3.1 Patient characteristics

**Figure 1:**
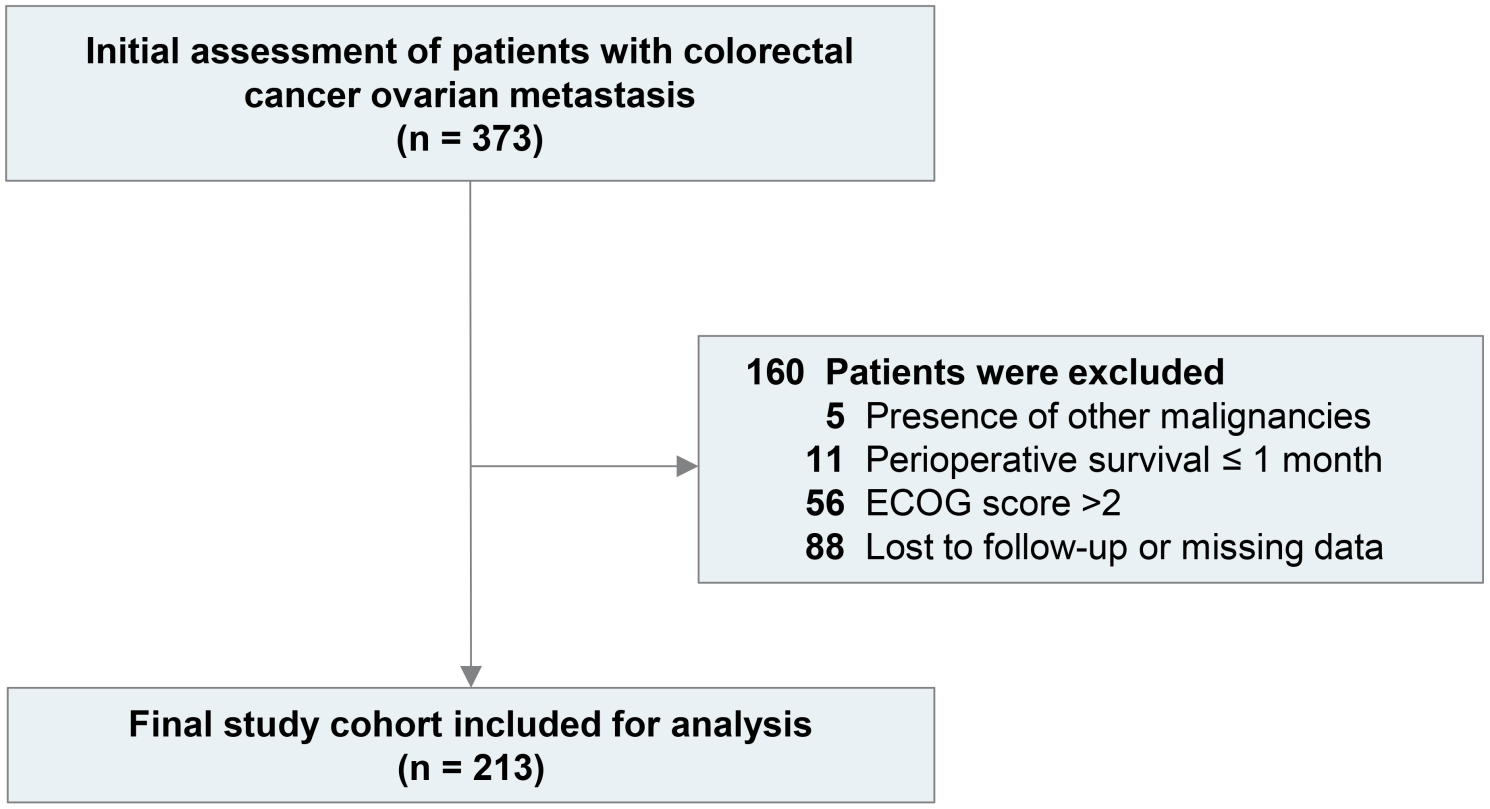
Patients’ selection process in this study.

Of the initial 373 patients with ovarian metastases from colorectal cancer, 213 were enrolled following the application of predetermined exclusion criteria. Exclusions comprised five patients with concomitant or prior malignancies, 11 due to perioperative mortality (≤ 1 month), 56 with an ECOG score > 2, and 88 owing to incomplete data or loss to follow-up (**Figure 1**). The clinical and pathological features of the patients are summarized in **Table 1**. The median age was 50 years (interquartile range 28–85 years) and 132 (61.9%) patients were premenopausal. A total of 192 patients (90.2%) underwent oophorectomy for metastasis, 205 (96.2%) underwent resection of the primary tumor, and 169 (83.6%) received chemotherapy for OM after oophorectomy. A total of 159 patients (74.7%) presented with symptoms at CROM diagnosis, including abdominal pain (61/213, 28.6%), abdominal distension (61/213, 28.6%), and vaginal bleeding (6/213, 2.8%), while the remaining patients (54/213, 26%) had no clinical symptoms.

**Table 1:** Clinical and pathological characteristics, stratified by receipt of oophorectomy.

Characteristic	No-oophorectomy	Oophorectomy	*P* value
	Number (%)	Number (%)	
Age			0.281
≤50 years	12 (5.6%)	86 (40.4%)	
>50 years	9 (4.2%)	106 (49.8%)	
Menstruation			0.631
Premenopause	12 (5.6%)	120 (56.3%)	
Postmenopause	9 (4.2%)	72 (33.8%)	
CEA			0.500
≤5 ug/L	5 (2.4%)	34 (16%)	
>5 ug/L	16 (7.5%)	157 (74.1%)	
CA199			0.626
≤37 U/ml	8 (3.8%)	63 (29.6%)	
>37 U/ml	13 (6.1%)	129 (60.6%)	
Tumor Location			0.615
Right colon	3 (1.4%)	53 (24.9%)	
Transverse colon	1 (0.5%)	11 (5.2%)	
Left colon	1 (0.5%)	16 (7.5%)	
Sigmoid colon	7 (3.3%)	52 (24.4%)	
Rectum	9 (4.2%)	60 (28.2%)	
AJCC of 8^th^ T stage			0.850
T1 stage	0 (0.0%)	1 (0.5%)	
T2 stage	2 (0.9%)	30 (14.2%)	
T3 stage	10 (4.7%)	78 (36.8%)	
T4 stage	9 (4.2%)	82 (38.7%)	
AJCC of 8^th^ N stage			0.537
N0 stage	7 (3.3%)	71 (33.3%)	
N1 stage	5 (2.3%)	61 (28.6%)	
N2 stage	9 (4.2%)	60 (28.8%)	
ECOG score			0.470
0	4 (1.9%)	24 (11.3%)	
1	11 (5.2%)	105 (49.3%)	
2	6 (2.8%)	63 (29.6%)	
Resection of colorectal primary			<0.001
Yes	5 (2.3%)	3 (1.4%)	
No	16 (7.5%)	189 (88.7%)	
Classification			0.468
Adenocarcinoma	12 (5.6%)	129 (60.6%)	
Mucinous carcinoma	6 (2.8%)	49 (23.0%	
Signet ring cell carcinoma	3 (1.4%)	14 (6.6%)	
Differentiation			0.456
Well	6 (2.8%)	48 (22.5%)	
Moderate	13 (6.1%)	112 (52.6%)	
Poor	1 (0.5%)	29 (13.6%)	
Undifferentiated	1 (0.5%)	3 (1.4%)	
Timing of metastasis			0.315
Synchronous	14 (6.6%)	106 (49.8%)	
Metachronous	7 (3.3%)	86 (40.4%)	
Side of metastasis			0.528
Unilateral	13 (6.1%)	105 (49.3%)	
Bilateral	8 (3.8%)	87 (40.8%)	
Abdominal dropsy			0.325
Yes	15 (7.0%)	116 (54.5%)	
No	6 (2.8%)	76 (35.7%)	
Symptom			0.116
Non-symptom	10 (4.8%)	44 (21.2%)	
Abdominal pain	3 (1.4%)	58 (27.9%)	
Abdominal distension	6 (2.9%)	55 (26.4%)	
Dyschezia	0 (0.0%)	16 (7.7%)	
Vaginal bleeding	0 (0.0%)	6 (2.9%)	
Others	1 (0.5%)	9 (4.3%)	
Extraovarian metastasis			0.478
Yes	14 (6.6%)	148 (69.8%)	
No	6 (2.8%)	44 (20.8%)	
Chemotherapy			0.082
Yes	14 (6.9%)	155 (76.7%)	
No	6 (3.0%)	27 (13.4%)	
VEGF			0.700
Positive	17 (8.0%)	143 (67.1%)	
Negative	4 (1.9%)	49 (23.0%)	
EGFR			0.902
Positive	12 (5.6%)	107 (50.2%)	
Negative	9 (4.2%)	85 (39.9%)	
P53			0.466
Positive	14 (6.6%)	107 (50.2%)	
Negative	7 (3.3%)	85 (39.9%)	
MMR			0.726
pMMR	20 (9.4%)	179 (84.0%)	
dMMR	1 (0.5%)	13 (6.1%)	

AJCC, American Joint Committee on Cancer; ECOG, Eastern Cooperative Oncology Group; EGFR, epidermal growth factor receptor; MMR, mismatch repair; VEGF, vascular endothelial growth factor.

The primary tumors were located in the colon in 144 patients (67.6%) and the rectum in 69 patients (32.4%). The depth of invasion of the primary cancer was T4 in 91 patients (42.9%). Lymph node metastases were detected in 135 patients (66.3%), and a total of 28 (13.1%) patients had an ECOG score of 0, 116 (54.5%) patients had a score of 1, and 69 (32.4%) patients had a score of 2. The study included 54 patients (25.3%) with well-differentiated tumors, 125 patients (58.7%) with moderately differentiated tumors, 30 patients (14.1%) with poorly differentiated tumors, and 4 patients (1.9%) with undifferentiated tumors. Overall, 141 patients (66.2%) had adenocarcinomas and 55 (25.8%) and 17 (8%) had mucinous carcinoma and signet ring cell carcinoma, respectively. Synchronous OM occurred in 120 patients (56.4%) and metachronous OM occurred in 93 patients (43.6%). The median interval from diagnosis of the primary to diagnosis of OM in the latter group was 15.7 months (range, 4–48 months). Among these patients, 95 (44.6%) developed bilateral OM and 87 (40.8%) underwent surgical treatment. Unilateral OM occurred in 118 patients (55.4%), of whom 105 (49.3%) underwent surgical treatment. The remaining 50 patients (23.6%) had simultaneous metastases to other organs, including peritoneal dissemination (13 patients, 6.1%), liver metastasis (27 patients, 12.7%), and lung metastasis (nine patients, 4.2%). A total of 159 patients (26%) presented with symptoms during their visit, including 122 (58.6%) presenting with abdominal pain or distension, 16 (7.7%) with dyschezia, and six (2.9%) with vaginal bleeding.

Regarding the molecular characteristics of the patients, 160 (75.1%) exhibited positive VEGF expression, 119 (55.9%) exhibited positive EGFR expression, 121 (56.8%) exhibited positive P53 expression, and 199 (93.4%) were characterized by a pMMR status.

### 3.2 Univariate survival and multivariate regression analyses

**Figure 2:**
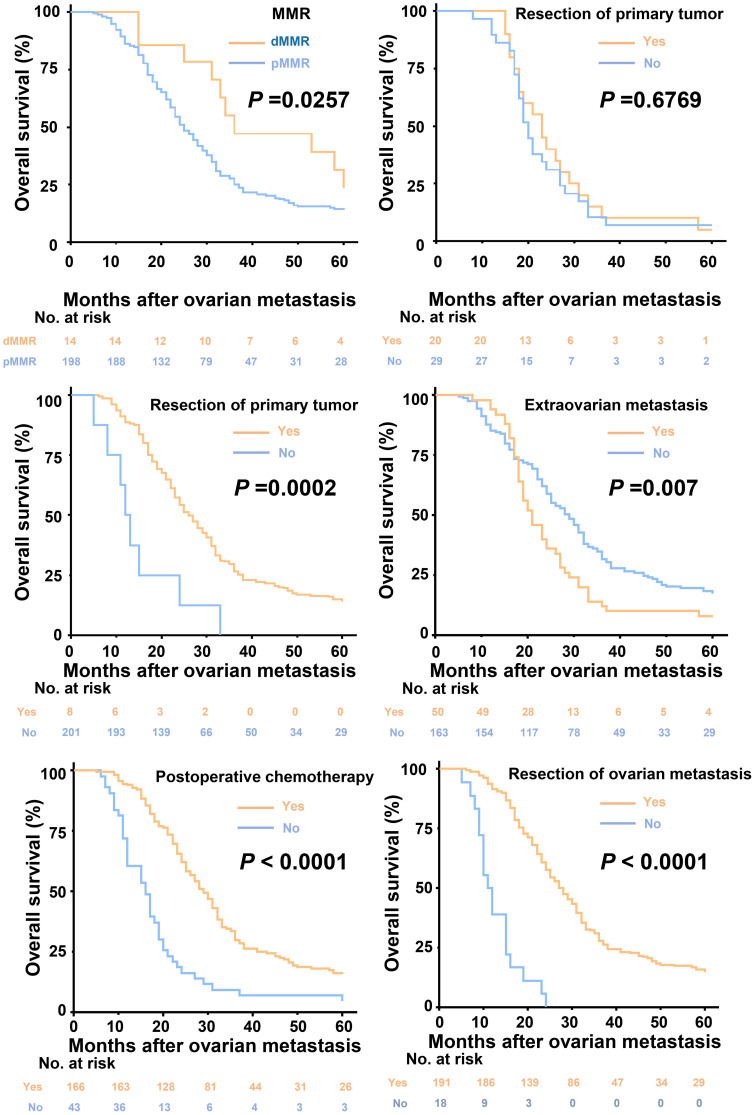
Kaplan-Meier estimates of overall survival in patients stratified by resection of primary tumor, extraovarian metastasis, oophorectomy, postoperative chemotherapy and mismatch repair status.

Univariate survival analysis was performed by the Kaplan-Meier method. The median OS after OM diagnosis was 23.0 months (95% CI: 16.0–32.0 months; **Figure 2**). The 5-year OS rate in patients treated by resection was 16.7% and the median survival time (MST) was 25.0 months (95% CI, 22.5–27.5 months, **Figure 2**). Among patients who did not undergo resection, the 5-year OS rate was 0% and the MST was 11 months (95% CI, 9.6–12.5 months, **Figure 2**). The 5-year OS rate among patients who underwent chemotherapy after surgery was 17.3% and the MST was 25.0 months (95% CI, 22.5–27.5 months, **Figure 2**), while the 5-year OS rate in patients without chemotherapy was 4% and the MST was 20.0 months (95% CI, 17.8–22.2 months, **Figure 2**). In patients with dMMR status, the 5-year OS rate was 28.57%, with a MST of 36 months (95% CI, 22.539-27.461 months, **Figure 2**). In contrast, patients with pMMR status had a 5-year OS rate of 14.57%, with a MST of 25 months (95% CI, 22.539-27.461 months, **Figure 2**). In patients with unresected extra-ovarian metastases, the 5-year OS rate was 6.67%, with a MST of 20 months (95% CI, 17.900–22.100 months; **Figure 2**). In contrast, among those who underwent resection of extra-ovarian metastases, the 5-year OS rate was 5%, and the MST was 23 months (95% CI, 18.639–27.361 months; **Figure 2**). The results of univariate survival analysis are shown in **Table 2**.

**Table 2:** Univariable and multivariable analyses of prognostic factors.

Variables	Univariable analysis		Multivariable analysis	
	HR (95% CI)	*P* value	HR (95% CI)	*P* value
Age	1.141 (0.847-1.537)	0.384		
≤50 years				
>50 years				
Menopausal status	1.100 (0.810-1.493)	0.541		
Postmenopause				
Premenopause				
CEA	0.761 (0.519-1.117)	0.163		
≤5 ug/L				
>5 ug/L				
CA199	1.027 (0.751-1.406)	0.865		
≤37 U/ml				
>37 U/ml				
Location	1.014 (0.759-1.428)	0.804		
Rectum				
Colon				
AJCC of 8^th^ T stage	1.176 (0.870-1.589)	0.291		
T1-3 stage				
T4 stage				
AJCC of 8^th^ N stage	0.877 (0.645-1.193)	0.405		
N0 stage				
N+ stage				
Resection of colorectal primary	3.157 (1.543-6.458)	0.002	1.103 (0.454-2.682)	0.828
Yes				
No				
Histology types	0.865 (0.677-1.105)	0.245		
Adenocarcinoma				
Mucinous carcinoma				
Signet ring cell cancer				
Differentiation	0.875 (0.590-1.299)	0.507		
Well/Moderate				
Poor/Undifferentiation				
Timing of ovarian metastasis	0.853 (0.632-1.151)	0.298		
Synchronous				
Metachronous				
Side of metastasis	1.011 (0.751-1.359)	0.945		
Unilateral				
Bilateral				
Abdominal dropsy	1.047 (0.775-1.414)	0.767		
Yes				
No				
Symptom	1.032 (0.934-1.140)	0.535		
Yes				
No				
Extra-ovarian metastasis	1.692 (1.199-2.386)	0.003	1.217 (0.834-1.776)	0.308
Yes				
No				
Resection of extra-ovarian metastasis	0.764 (0.419-1.391)	0.379		
Yes				
No				
Oophorectomy	15.451 (9.018-26.473)	<0.001	10.476 (5.536-19.825)	<0.001
Yes				
No				
Postoperative chemotherapy	6.156 (3.895-9.731)	<0.001	2.232 (1.538-3.238)	<0.001
Yes				
No				
VEGF	0.849 (0.604-1.193)	0.344		
Positive				
Negative				
EGFR	1.181 (0.881-1.584)	0.265		
Positive				
Negative				
P53	1.149 (0.856-1.542)	0.356		
Positive				
Negative				
MMR	1.831 (0.967-3.469)	0.064	1.967 (1.026-3.772)	0.042
pMMR				
dMMR				

AJCC, American Joint Committee on Cancer; EGFR, epidermal growth factor receptor; HR, hazard ratio; MMR, mismatch repair; VEGF, vascular endothelial growth factor.

Multivariate Cox proportional hazard regression analysis showed that oophorectomy (HR = 10.476, 95% CI, 5.536–19.825; P < 0.001), chemotherapy after surgery (HR = 2.232, 95% CI, 1.538–3.238; P < 0.001) and MMR status (HR = 1.967, 95% CI, 1.026-3.772; P = 0.042) were independent prognostic factors in CROM patients. The details are presented in **Table 2**.

### 3.3 Stratified analyses

The results of stratified analyses indicated that, within each subgroup, oophorectomy and postoperative chemotherapy were prognostic factors for ovarian metastasis of colorectal cancer. Additionally, MMR status emerged as a significant prognostic factor in populations with colon as the primary site, lymph node metastasis, adenocarcinoma, mucinous adenocarcinoma and signet-ring cell carcinoma. 

## 4 Discussion

The incidence of CROM is low and there are currently no reference guidelines for its treatment. The current study including 213 patients with CROM indicated that oophorectomy, postoperative chemotherapy and dMMR status were independent prognostic factors associated with better OS.

Previous studies have investigated several prognostic factors for ovarian metastasis in colorectal cancer (8, 13, 19, 20). In comparison, this study offers more notable advancements. Firstly, while earlier studies typically had fewer than 100 cases, this study includes 213 which significantly increasing statistical power and enhancing result reliability. Secondly, previous studies largely focused on the correlation between clinical/pathological features and prognosis, without a systematic analysis of molecular characteristics. This study evaluated the prognostic impact of immunohistochemical markers such as VEGF, EGFR, P53, and MMR, offering a more comprehensive analysis. Additionally, it is the first to identify dMMR status as a favorable prognostic factor for ovarian metastasis in colorectal cancer.

dMMR results in the failure to repair mismatched DNA, causing microsatellite instability (MSI) characterized by a high burden of insertions and deletions. A meta-analysis of 31 studies involving 7,642 CRC patients, including 1,277 MSI-positive cases, demonstrated a 35% reduced risk of death for MSI tumors compared to microsatellite stable (MSS) tumors (21). This improved prognosis is largely due to the lower recurrence rates observed in stage II and III MSI CRCs (22, 23). Biologically, dMMR induces genomic instability, generating neoantigens that activate T cell-mediated immune responses, enhancing immune surveillance and inhibiting tumor growth and metastasis. Additionally, dMMR tumors typically exhibit higher mutational burdens, making them more recognizable to the immune system and more responsive to immune checkpoint inhibitors such as PD-1/PD-L1 inhibitors. This loss of immune evasion further improves the effectiveness of immunotherapy, leading to better patient outcomes. In the tumor microenvironment, dMMR tumors are often associated with increased immune cell infiltration, particularly active CD8+ T cells, which may help suppress metastatic spread (24). However, the prognostic value of MSI status in metastatic settings remains debated. Although our study identifies dMMR as a favorable prognostic factor, it is important to note that the dMMR population constitutes only 6.6% of the study cohort. Therefore, these findings require further validation through additional research in the future.

Patients with CROM often have no obvious symptoms; however, increases in the size of the metastatic tumor result in corresponding compression symptoms, including abdominal pain (50.5%–64.3%) and distension (7.8%–35.7%) as the most common symptoms (10, 25). About 40% of patients in a previous study were asymptomatic at the time of treatment (26), compared with about 26% at the time of treatment in the current study, which may explain why they failed to detect the disease in time. A retrospective study in 2010 evaluated the treatment response in 33 patients who received chemotherapy and found that OM was significantly less responsive to chemotherapy compared with metastases outside the ovary. (18.2% vs 33.3%) (19). Twenty-three patients received chemotherapy before oophorectomy, of whom 15 had extraovarian metastases under control (objective remission or stability), and eight patients (35%) had disease progression. In contrast, no remission of OM was observed, with three patients (13%) having stable disease and progression in 20 patients (87%). The chemotherapy responses of patients with OM and metastases at other sites were significantly different (14). The mechanism of drug resistance in OM tumors is unclear, but some studies have suggested that it might be associated with gene mutations (such as *KRAS*, *NRAS*, *BRAF*) (27, 28); however, the scale of this study was relatively small and the results need further verification.

CEA is an adhesion molecule that promotes the aggregation of colorectal cancer cells and is often used as a postoperative tumor marker; however, information on the value of CEA for detecting CROM is lacking. In a study in 2010, 13 of 20 patients (72.2%) had elevated CEA levels and nine of 20 (45.0%) had elevated CA19–9 levels. Ten of 13 patients (76.9%) with metachronous OM had elevated CEA levels, and CA19–9 was elevated in seven of 13 patients (53.8%) (29). A retrospective study conducted in 2020 included 46 patients, of whom 29 (63.0%) had elevated CEA at presentation and 17 (37.0%) were within the normal range (13). In the present study, 173 (81.2%) and 142 patients (66.7%) had elevated CEA and CA199 levels, respectively, at the time of diagnosis of OM, but this was not identified as an independent prognostic factor. Although CEA and CA199 have been widely used in the prediction and prognosis of liver metastasis of colorectal cancer, their role in the detection of CROM needs further verification (6, 30, 31). Several small retrospective clinical studies have explored the prognostic impact of oophorectomy in patients with CROM. One retrospective study involving 130 patients showed that the survival time was significantly longer in patients in the oophorectomy group compared with the non-resection group (28.1 vs 21.2 months), and oophorectomy was an independent prognostic factor (19). A retrospective Japanese study including 30 patients found a median survival time after oophorectomy of about 34.9 months and a 3-year OS rate of 51.1%, while the median survival in patients with peritoneal metastasis was only 21.0 months; however, the sample size was small and there may have been large bias (15). Chen et al. proposed a scoring system to identify patients with OM who were less likely to benefit from surgical treatment, to inform preoperative and intraoperative decision making and to judge the survival benefit of surgery (32). Future treatment strategies for patients with CROM should be screened carefully to identify those likely to benefit from surgical removal. These findings were in accord with the current findings, suggesting that oophorectomy can improve OS in patients with CROM.

Because the ovary acts as a refuge for metastatic substances, 87% of cases of CROM were reported to show tumor progression or new metastases during chemotherapy, compared with metastatic organs outside the ovaries (liver, lung, peritoneum) (14, 28). In contrast to previous findings, however, the above study found that use of systemic adjuvant chemotherapy was an independent predictor of improved OS (10). The median survival of patients receiving adjuvant chemotherapy was significantly longer than that of patients without adjuvant chemotherapy (28.8 vs 8.2 months) (8). Among 57 patients with CROM who underwent surgery, systemic chemotherapy was significantly associated with a better prognosis.

This study had some limitations. First, it is important to acknowledge the inherent limitations of our study, particularly the potential for unmeasured confounding and selection bias due to the retrospective design. These factors may limit the generalizability of our findings. Second, the study duration was long, in order to collect more cases, and the treatment scheme may thus be biased. The other limitation is the substantial imbalance in sample sizes between the oophorectomy group and the no oophorectomy group, which may introduce bias in the multivariate analysis. This disparity could affect the precision of the effect estimates. Future multi-center, large-scale, prospective studies are needed to better control for confounders, reduce bias, and further validate the results.

## 5 Conclusions

Oophorectomy, postoperative chemotherapy and dMMR status may provide survival benefits for colorectal cancer patients with ovarian metastasis; however, the main findings from current study may require further validations.

## Data Availability

The raw data supporting the conclusions of this article will be made available by the authors, without undue reservation.
